# Incidence and Time to Onset of Pseudophakic Cystoid Macular Edema (Irvine-Gass Syndrome) After 1,325 Consecutive Cataract Surgeries Performed by a Single Surgeon Over Four Years

**DOI:** 10.7759/cureus.114802

**Published:** 2026-08-19

**Authors:** Toshihiko Matsuo, Chie Nakago-Matsuo

**Affiliations:** 1 Ophthalmology, Ochiai Hospital, Maniwa, JPN; 2 Ophthalmology, Graduate School of Interdisciplinary Science and Engineering in Health Systems, Okayama, JPN; 3 Orthodontics, Graduate School of Environmental, Life, Natural Science and Technology, Okayama University, Okayama, JPN

**Keywords:** betamethasone, bromfenac, irvine-gass syndrome, macular cyst, moxifloxacin, optical coherence tomography, pseudophakic cystoid macular edema, single surgeon, small-incision cataract surgery, topical medication

## Abstract

Objectives

Macular edema after cataract surgery is known as pseudophakic cystoid macular edema or Irvine-Gass syndrome. The aim of this study was to determine the incidence and time to onset of cystoid macular edema after uncomplicated cataract surgery.

Methods

A retrospective review was conducted of 1,325 consecutive cataract surgeries performed by a single surgeon at a single institution over four years, from January 2022 to December 2025.

Results

Among 1,325 eyes, one or more macular cysts were detected by optical coherence tomography in 27 eyes (2.0%) of 22 patients who complained of blurred vision or decreased visual acuity after having no symptoms immediately after surgery. The 22 patients (27 eyes: 12 right and 15 left) comprised 12 men and 10 women. Their ages at the time of surgery ranged from 57 to 82 years, with a median of 75.5 years. Of the 27 eyes that developed macular cysts, 13 eyes with no preoperative or intraoperative risk factors for inflammation received topical 0.1% bromfenac twice daily as the only postoperative anti-inflammatory treatment, together with topical antibacterial 0.5% moxifloxacin four times daily. In contrast, the remaining 14 eyes, which had risk factors for inflammation, such as exfoliation, floppy iris, poor mydriasis, extracapsular cataract extraction, or diabetes mellitus, received topical bromfenac twice daily and 0.1% betamethasone (or 0.1% fluorometholone in two eyes) four times daily, as well as topical moxifloxacin. The time from surgery to the diagnosis of macular cysts ranged from one week to eight months, with a median of two weeks, in the 13 eyes treated with topical bromfenac only. In the 14 eyes treated with the combination of topical bromfenac and betamethasone, the time to diagnosis ranged from two weeks to 13 months, with a median of two months. In 26 of the 27 eyes, the macular cysts disappeared and visual acuity returned to normal without residual symptoms within two weeks to seven months, with a median of one month, after topical betamethasone four times daily was initiated or resumed.

Conclusions

Pseudophakic macular cysts developed during the postoperative course in eyes with no preoperative or intraoperative risk factors that received topical bromfenac alone. They also developed later in the postoperative course after the combination of topical bromfenac and betamethasone was discontinued in eyes with risk factors for inflammation. The macular cysts disappeared following the initiation or resumption of topical betamethasone, accompanied by recovery of visual acuity and resolution of symptoms.

## Introduction

The macula has a unique architecture characterized by a high density of photoreceptor cells and plays a crucial role in high-resolution vision. Macular edema is the intraretinal or subretinal accumulation of fluid that disrupts the normal retinal architecture and causes visual symptoms such as metamorphopsia, blurring, and decreased visual acuity [1]. Pseudophakic cystoid macular edema, also called Irvine-Gass syndrome, is macular edema that occurs after cataract surgery with intraocular lens implantation [2, 3, 4]. Macular edema after cataract surgery was initially reported in the era of extracapsular or intracapsular cataract extraction, when intraocular lenses were not yet widely used, in the 1950s and 1960s [5-7]. Later, in the 1980s, cataract surgery using phacoemulsification and intraocular lens implantation became the standard treatment, and in the 1990s, foldable intraocular lenses made of acrylic polymers became available, enabling modern small-incision cataract surgery.

Even in the modern era of small-incision cataract surgery, pseudophakic cystoid macular edema appears to occur in a few percent of uncomplicated cataract surgeries [2]. Macular examination using optical coherence tomography through non-mydriatic, normal-sized pupils has become standard in current ophthalmic practice, facilitating the accurate diagnosis of macular edema [8, 9]. In general, macular edema is associated with diabetic retinopathy, branch or central retinal vein occlusion, and neovascular age-related macular degeneration [10]. Macular edema has also rarely been associated with topical prostaglandin analogues, such as latanoprost, used to lower intraocular pressure in the treatment of glaucoma [11-13]. The term Irvine-Gass syndrome generally does not include the onset or exacerbation of macular edema after cataract surgery when it is attributable to preexisting retinal diseases such as diabetic retinopathy or retinal vein occlusion. An epiretinal membrane is also a known risk factor for developing macular edema after cataract surgery [14].

Because postoperative inflammation is minimal after modern small-incision cataract surgery, the routine postoperative administration of topical corticosteroid eye drops is often omitted. Instead, topical nonsteroidal anti-inflammatory drug (NSAID) eye drops are used as part of the routine postoperative regimen together with topical antibacterial eye drops [15, 16]. Topical corticosteroid eye drops are added to this postoperative regimen in cases involving operative complications or preexisting risk factors for postoperative intraocular inflammation [17]. Well-known risk factors for postoperative intraocular inflammation include poorly controlled diabetes mellitus, even without retinopathy; retinitis pigmentosa; exfoliation syndrome; and a history of uveitis or blunt ocular trauma [18-20]. In this study, we investigated the incidence and timing of the onset of pseudophakic cystoid macular edema following consecutive cataract surgeries performed by a single surgeon at a single institution over a four-year period.

## Materials and methods

Study design

This retrospective study involved 1,325 consecutive cataract surgeries performed as either day surgery or overnight-stay surgery by a single surgeon (Toshihiko Matsuo) from January 2022 to December 2025 at Ochiai Hospital, a local hospital with 110 beds in Maniwa City, which had a population of approximately 39,000 in 2026 and is located in the northern part of Okayama Prefecture, Japan. Day surgery and overnight-stay surgery each accounted for approximately half of the surgeries, and the type of surgery was selected solely according to the patients’ preferences. The study was approved as a retrospective observational study by the Ethics Committee of Okayama University Graduate School of Medicine, Dentistry, and Pharmaceutical Sciences and Okayama University Hospital (identifier: 2502-001; date of approval: November 27, 2024) and by the Ethics Committee of Ochiai Hospital. Medical records were reviewed to collect data on age, sex, systemic and ocular diseases, postoperative use of topical corticosteroid eye drops, postoperative macular edema detected by optical coherence tomography, and best-corrected visual acuity.

Surgical procedures and care

All surgeries were performed under local anesthesia, usually using topical 4% lidocaine eye drops alone or in combination with a sub-Tenon injection of 2% lidocaine. Most cataract surgeries involved standard phacoemulsification and aspiration with intraocular lens implantation through a 2.4-mm-wide superior corneal incision. In cases involving thin and weak lens capsules in mature or brown cataracts, extracapsular cataract extraction was planned and performed, with implantation of an intraocular lens fixated in the capsular bag. When the capsular bag could not be preserved because of weak ciliary zonules, the intraocular lens was sutured in place using a long, curved needle with a 10-0 polypropylene suture (PC-9, Alcon, Geneva, Switzerland) [21]. Before surgery, all patients underwent electrocardiography, plain chest radiography, and blood examinations, including complete blood cell counts, blood chemistry screening tests, and blood glucose measurements.

As a routine procedure, conjunctival sac bacterial cultures were performed in all patients, usually one month before surgery, and antimicrobial susceptibility testing was performed when bacterial species were detected [17]. Topical 0.5% moxifloxacin eye drops were administered four times daily from three days before surgery through the day of surgery. When bacterial species resistant to moxifloxacin were detected, 0.5% cefmenoxime eye drops were administered from three days before surgery if the bacterial species were susceptible to cephems. When bacterial species were resistant to both moxifloxacin and cephems, as in cases involving methicillin-resistant *Staphylococcus aureus* or *Staphylococcus epidermidis*, topical 0.5% arbekacin eye drops prepared from an injectable solution (100 mg/2 mL) were administered for one week, and a repeat conjunctival sac culture was performed to confirm a negative culture result. The main purpose of routinely performing conjunctival sac cultures in all patients before cataract surgery was to prevent the introduction of methicillin-resistant *Staphylococcus aureus* or *Staphylococcus epidermidis* into the hospital [17, 22].

During surgery, the ocular surface was frequently irrigated with a 20-fold saline dilution of 10% povidone-iodine, resulting in a working concentration of 0.5%. In patients with an iodine allergy, 0.1% chlorhexidine was used instead to disinfect the skin, and saline-diluted chlorhexidine at a working concentration of 0.02% was used to irrigate the ocular surface before and during surgery [17]. After surgery, topical 0.5% levofloxacin, 0.1% betamethasone ointment, and 0.3% ofloxacin ointment were applied to the ocular surface. A gauze eye patch was worn overnight and removed the following morning. In patients with poor or no vision in the unoperated fellow eye, protective transparent plastic glasses were used instead of a gauze eye patch following the instillation of topical 0.5% levofloxacin alone.

On the day after surgery, all patients started using a new 5-mL bottle of 0.5% moxifloxacin four times daily, usually for two weeks, in combination with 0.1% bromfenac twice daily for one month. Topical 0.1% betamethasone four times daily was additionally prescribed in cases involving conditions associated with an increased risk of inflammation, such as floppy iris syndrome, exfoliation syndrome, poor mydriasis, extracapsular cataract extraction, intraocular lens suturing, retinitis pigmentosa, poorly controlled diabetes mellitus without retinopathy, and a history of uveitis or iritis. All patients attended routine follow-up visits on the day after surgery and at five days, one week, and two weeks after surgery to check for complications such as iritis and a transient increase in intraocular pressure. Follow-up was usually discontinued two weeks after surgery when there were no concerns regarding inflammation, intraocular pressure, or visual acuity.

## Results

Table 1 summarizes the clinical features of the retrospective cohort of consecutive cataract surgeries performed by a single surgeon at a single institution. During the four-year period from 2022 through 2025, 1,325 cataract surgeries were performed in 668 (50.4%) right eyes and 657 (49.6%) left eyes of 827 patients, comprising 375 (45.3%) men and 452 (54.7%) women. The patients’ ages at the time of their first surgery ranged from 43 to 101 years, with a median of 75 years and a mean of 75.5 years. The number of operated eyes in patients with diabetes mellitus was 305 (23.0% of the total 1,325 eyes). Topical corticosteroid eye drops, mostly 0.1% betamethasone, were used in addition to routine topical 0.1% bromfenac during the immediate postoperative period in 597 of the 1,325 operated eyes (45.0%). Topical bromfenac alone was used as the anti-inflammatory eye drop in the remaining 728 operated eyes (54.9%). Sixty-eight eyes (5.1%) were operated on in patients who were taking α1-adrenergic blockers for benign prostatic hyperplasia, which is a risk factor for intraoperative floppy iris syndrome and associated inflammation.

**Table 1:** Systemic conditions and reasons for the postoperative administration of topical corticosteroid eye drops in combination with topical nonsteroidal anti-inflammatory drug (NSAID) eye drops following cataract surgery in 1,325 consecutive eyes over four years (2022-2025) *Corticosteroid: predominantly topical 0.1% betamethasone and, rarely, topical 0.1% fluorometholone. Mydrin-P: a topical combination of 0.5% tropicamide and 0.5% phenylephrine hydrochloride.

Category or treatment	Clinical feature or indication	Number of eyes (%)
Systemic conditions	Patients taking α1-adrenergic blockers for benign prostatic hyperplasia	68 (5.1%)
	Patients undergoing hemodialysis	32 (2.4%)
	Patients with diabetes mellitus	305 (23.0%)
Postoperative corticosteroid use	Eyes receiving topical corticosteroid* eye drops postoperatively	597 (45.1%)
Reasons for postoperative topical corticosteroid* eye drops		
Topical 0.1% fluorometholone instead of topical 0.1% bromfenac		
Systemic diseases	Rheumatoid arthritis	10 (0.75%)
	Polymyalgia rheumatica	3 (0.23%)
Topical 0.1% betamethasone instead of topical 0.1% bromfenac		
Systemic diseases	Rheumatoid arthritis	7 (0.53%)
	Polymyalgia rheumatica	4 (0.30%)
Topical 0.1% betamethasone in combination with topical 0.1% bromfenac		
Systemic diseases	Graves disease	1 (0.075%)
Retinal and intraocular conditions	Diabetic retinopathy	29 (2.2%)
	Clinically significant epiretinal membrane	3 (0.23%)
	History of branch retinal vein occlusion	2 (0.15%)
	History of uveitis	18 (1.4%)
	Retinitis pigmentosa	3 (0.23%)
Ocular surface and surgical history	History of scleritis	2 (0.15%)
	Previous LASIK (laser-assisted in situ keratomileusis)	3 (0.23%)
	History of blunt ocular trauma	3 (0.23%)
	Previous trabeculectomy	2 (0.15%)
	History of allergy to topical mydriatics (Mydrin-P)	2 (0.15%)
Iris and lens conditions	Exfoliation syndrome	27 (2.0%)
	Mature or brown cataract	16 (1.2%)
	Unstable ciliary zonules	35 (2.6%)
	Poor mydriasis	69 (5.2%)
	Floppy iris	73 (5.5%)
Intraoperative reasons	Extracapsular cataract extraction with intraocular lens implantation	23 (1.7%)
	Intraocular lens suturing	17 (1.3%)
	Trabeculotomy combined with cataract surgery	10 (0.75%)
	Pterygium resection combined with cataract surgery	18 (1.4%)
Postoperative reasons	A small number of keratic precipitates or aqueous cells, or Descemet membrane folds, on the day after surgery	193 (14.6%)
Other or unspecified reasons	Use of the same regimen as in the fellow eye	24 (1.8%)

The reasons for using topical 0.1% betamethasone in addition to topical 0.1% bromfenac were broadly classified into three categories: preoperative, intraoperative, and postoperative indications (Table 1). The combination of topical betamethasone and bromfenac was scheduled preoperatively in the presence of factors associated with an increased risk of inflammation, including exfoliation syndrome, poor mydriasis, and poorly controlled diabetes mellitus without retinopathy; a history of uveitis or blunt ocular trauma; and planned extracapsular cataract extraction with intraocular lens implantation for mature or brown cataracts. The intraoperative factors included floppy iris, ciliary zonules that were weaker than expected, and conversion to intracapsular cataract extraction with intraocular lens suturing. The postoperative indications for the addition of topical betamethasone were signs observed on the day after cataract surgery, including mild anterior chamber inflammation, such as keratic precipitates, Descemet membrane folds, and aqueous cells, in contrast to the absence of inflammation usually observed on the day after surgery in other patients. Other unspecified reasons for the additional use of topical betamethasone eye drops included the use of the same regimen as that used for the fellow eye following an earlier cataract surgery. The omission of topical 0.1% bromfenac and the use instead of topical 0.1% betamethasone or 0.1% fluorometholone alone were planned preoperatively in patients with rheumatoid arthritis because their corneas may be structurally weak and, although rarely, susceptible to the devastating complication of corneal melting. During the first half of the study period, surgeries in patients with polymyalgia rheumatica also followed this postoperative medication protocol.

Figures 1-13 show cross-sectional optical coherence tomography images from all 27 eyes of the 22 patients who developed macular edema after cataract surgery. The serial images demonstrate the course from the onset to the remission of pseudophakic cystoid macular edema, which varied in severity from a single macular cyst to a few cysts and, in the most severe cases, extensive macular edema resulting from the fusion of macular cysts.

**Figure 1:**
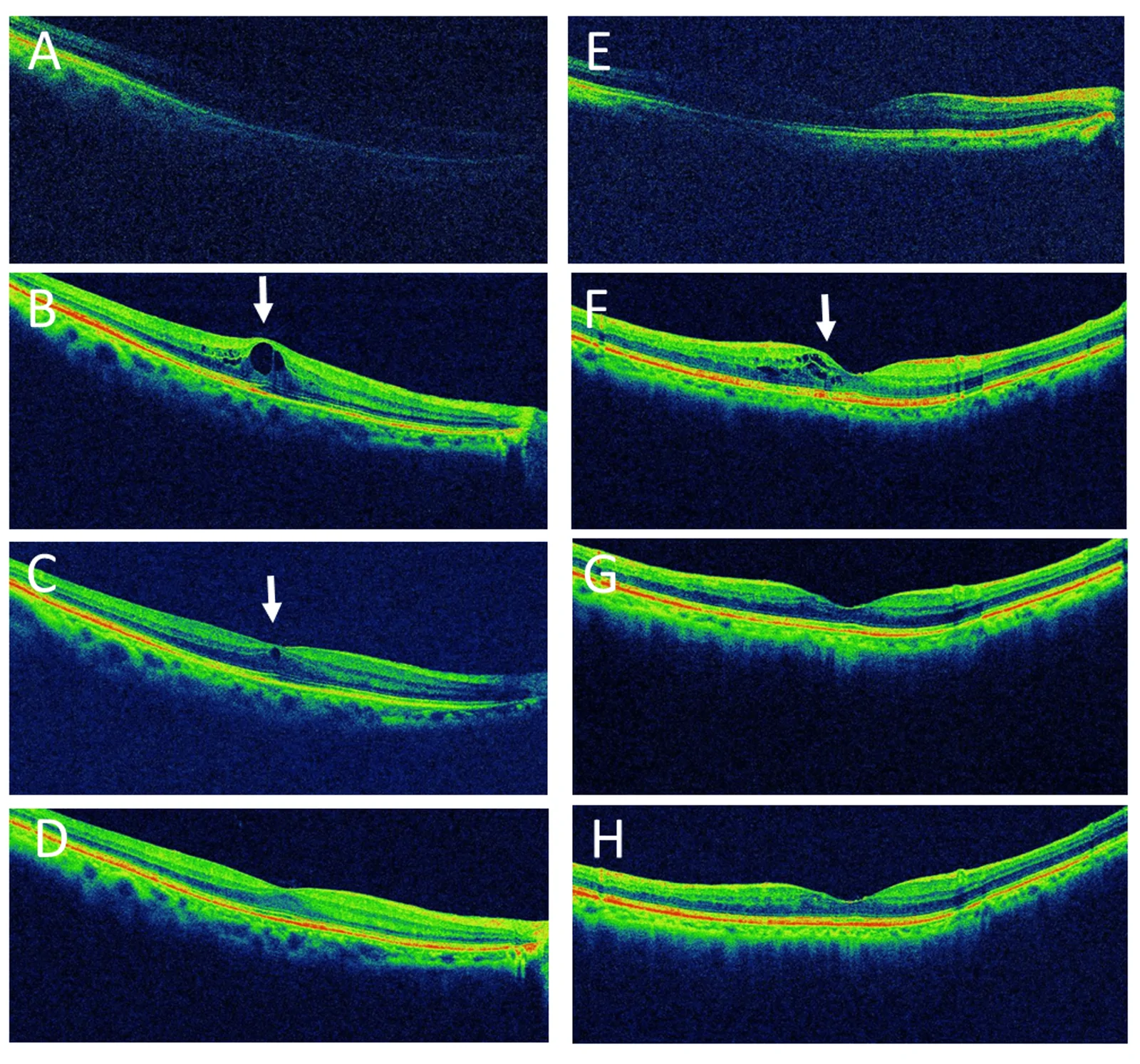
Optical coherence tomography findings in Cases 1 and 2. Case 1. Right eye in a 74-year-old man (A-D, left column). Horizontal section images of optical coherence tomography, showing no macular cyst before cataract surgery (A) and macular cysts 2 weeks after surgery with topical 0.1% bromfenac only (arrow, B). A small cyst 1 month (arrow, C) and no cyst 2 months (D) after topical 0.1% betamethasone use. Case 2: Right eye of a 73-year-old man (E-H, right column). Vertical cross-sectional optical coherence tomography images show no macular cyst before cataract surgery (E) and macular cysts two months after surgery following postoperative treatment with topical 0.1% bromfenac and 0.1% betamethasone (arrow, F). No macular cyst was observed at 1.5 months (G) or five months (H) after the resumption of topical 0.1% betamethasone. Images A and E are blurred because of the presence of cataracts before surgery.

**Figure 2:**
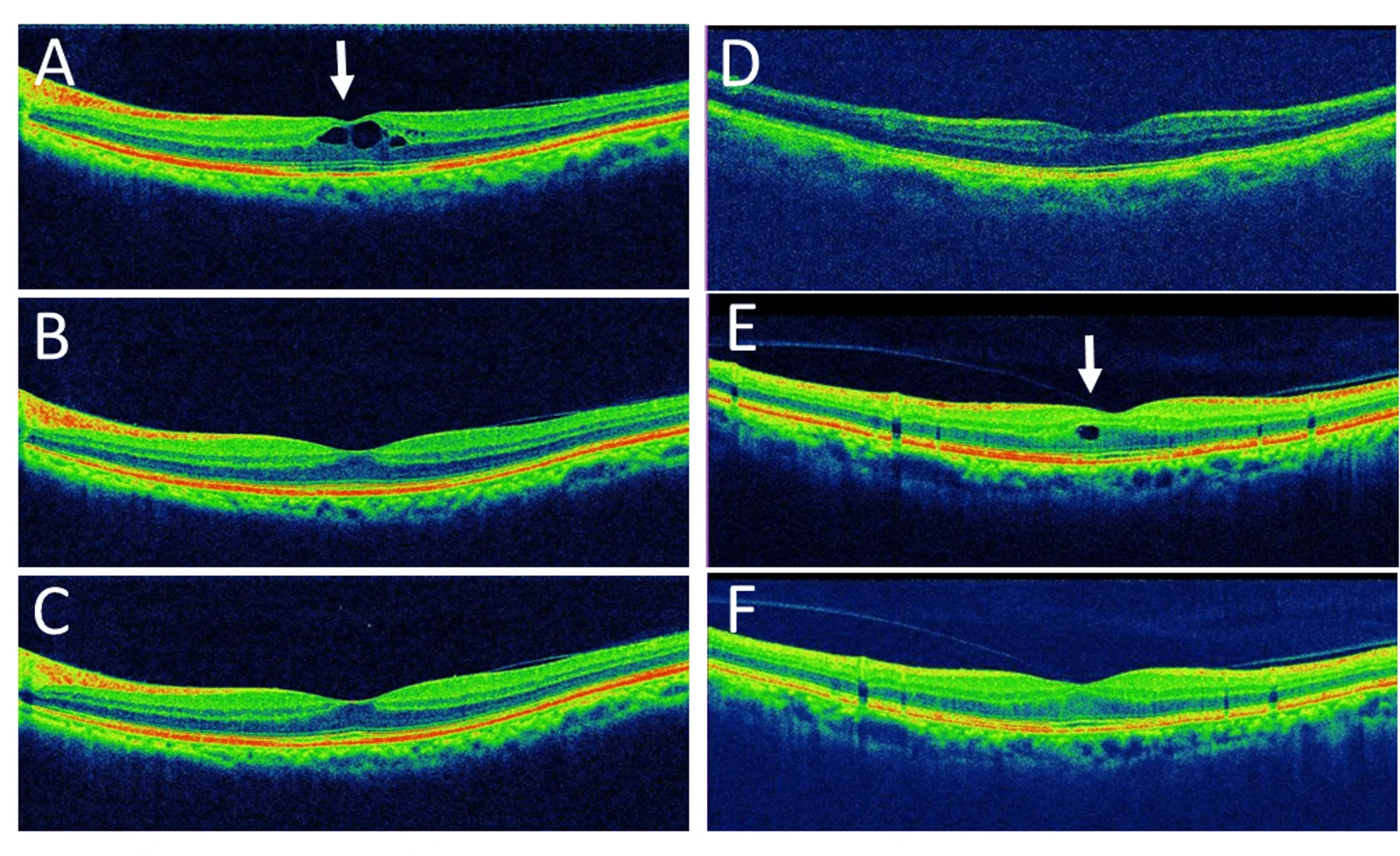
Optical coherence tomography in Cases 3 and 21. Case 3: Left eye of a 72-year-old woman (A-C, left column). Horizontal cross-sectional optical coherence tomography images show macular cysts one month after surgery while receiving topical 0.1% bromfenac alone (arrow, A). No cyst was observed at three weeks (B) or six weeks (C) after the initiation of topical 0.1% betamethasone. Case 21: Left eye of a 72-year-old woman (D-F, right column). Vertical cross-sectional optical coherence tomography images show no macular cyst before cataract surgery (D), a cyst two weeks after cataract surgery while receiving topical 0.1% bromfenac alone (arrow, E), and no cyst two weeks after the initiation of topical 0.1% betamethasone (F).

**Figure 3:**
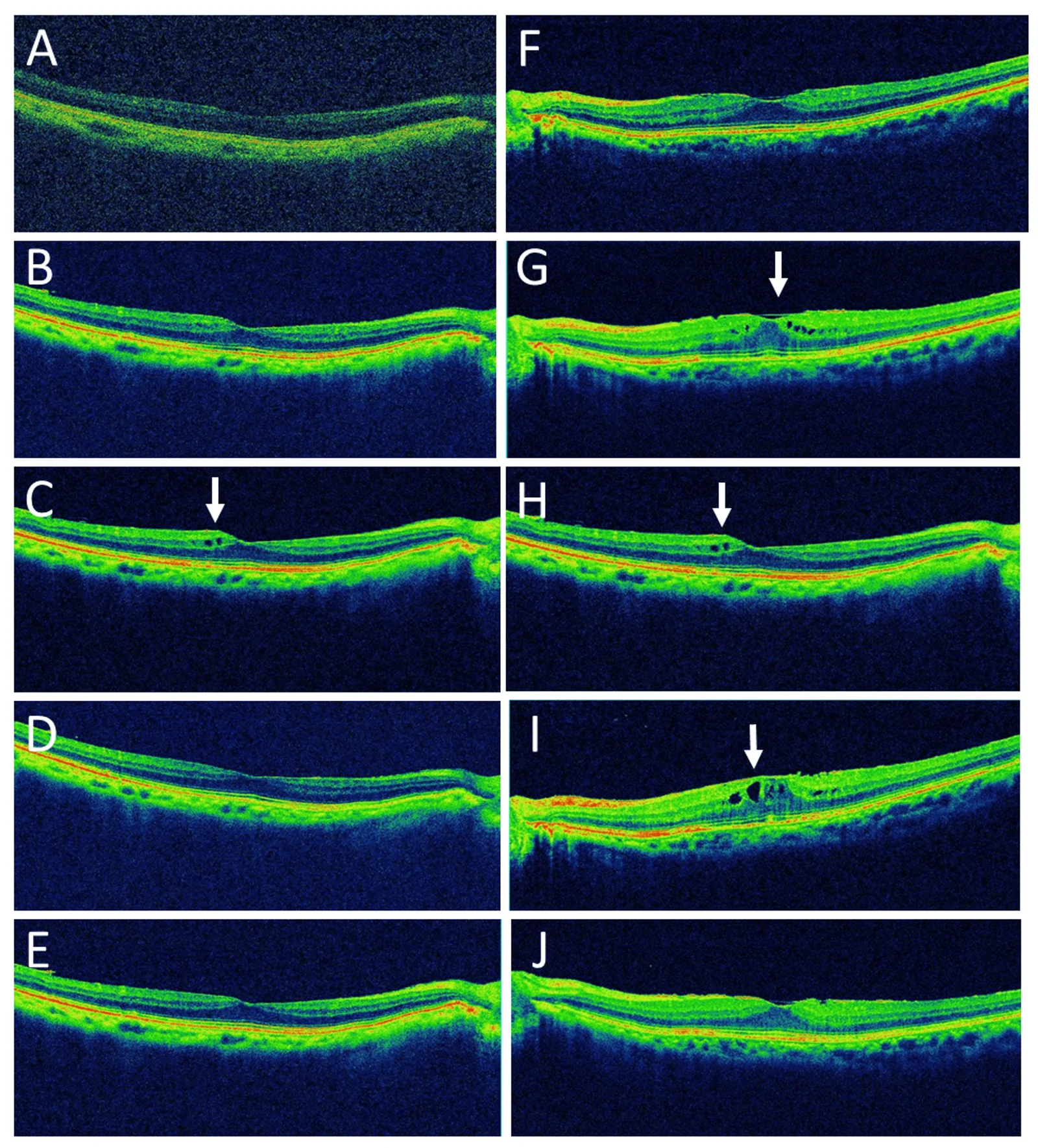
Optical coherence tomography in Case 4. Case 4: Right eye (A-E, left column) and left eye (F-J, right column) of a 74-year-old woman. Cataract surgery was first performed on the right eye and, one month later, on the left eye. Each pair of images in the same row was obtained on the same date. Horizontal cross-sectional optical coherence tomography images show no macular cyst in the right eye at five days (A) or one month (B) after extracapsular cataract extraction and intraocular lens implantation with topical 0.1% bromfenac and 0.1% betamethasone. Macular cysts were observed in the right eye two months after surgery (arrow, C). No cyst was observed at one month (D) or three months (E) after the resumption of topical 0.1% betamethasone. In the left eye, no cyst was observed before surgery (F), whereas macular cysts were observed 14 days after standard cataract surgery with topical 0.1% bromfenac and 0.1% betamethasone (arrow, G). Macular cysts remained at one month (arrow, H) and two months (arrow, I) after the resumption of topical 0.1% betamethasone, but no cyst was observed at four months (J). Note the minimal epiretinal membrane in the left eye.

**Figure 4:**
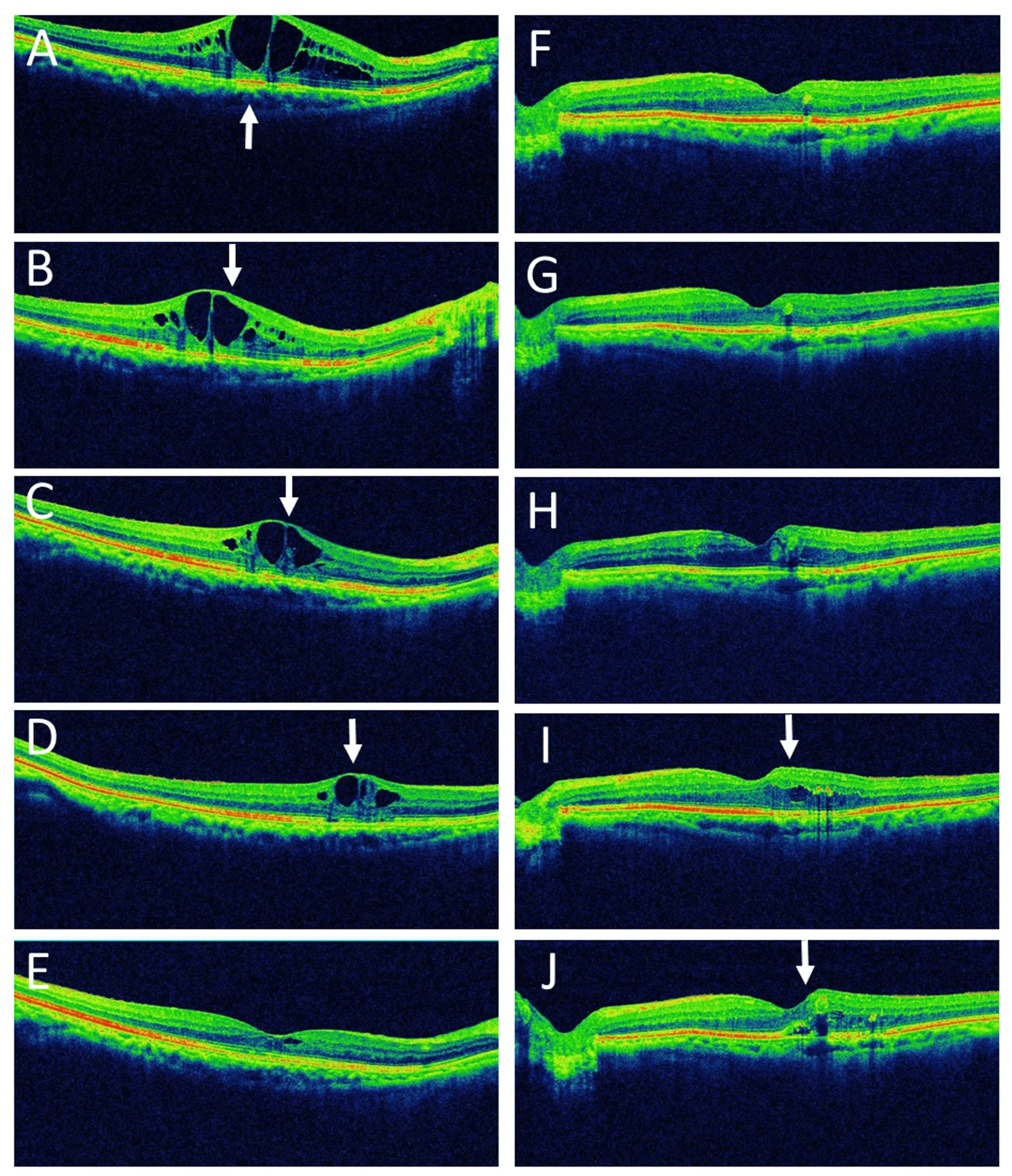
Optical coherence tomography in Cases 5 and 6. Case 5: Right eye of a 78-year-old woman (A-E, left column). Horizontal cross-sectional optical coherence tomography images show macular edema eight months after surgery while receiving topical 0.1% bromfenac alone (arrow, A). The macular edema gradually decreased at one month (arrow, B), three months (arrow, C), and five months (arrow, D) after the initiation of topical 0.1% betamethasone, with no cyst observed at seven months (E). Case 6: Left eye of a 74-year-old man (F-J, right column). Horizontal cross-sectional optical coherence tomography images show no macular cyst at four months (F), seven months (G), or 10 months (H) after surgery and postoperative treatment with topical 0.1% bromfenac and 0.1% fluorometholone. Macular cysts were observed 13 months after surgery (arrow, I). One month after the initiation of topical 0.1% betamethasone, no cyst was observed, although the foveal contour remained deformed (arrow, J).

**Figure 5:**
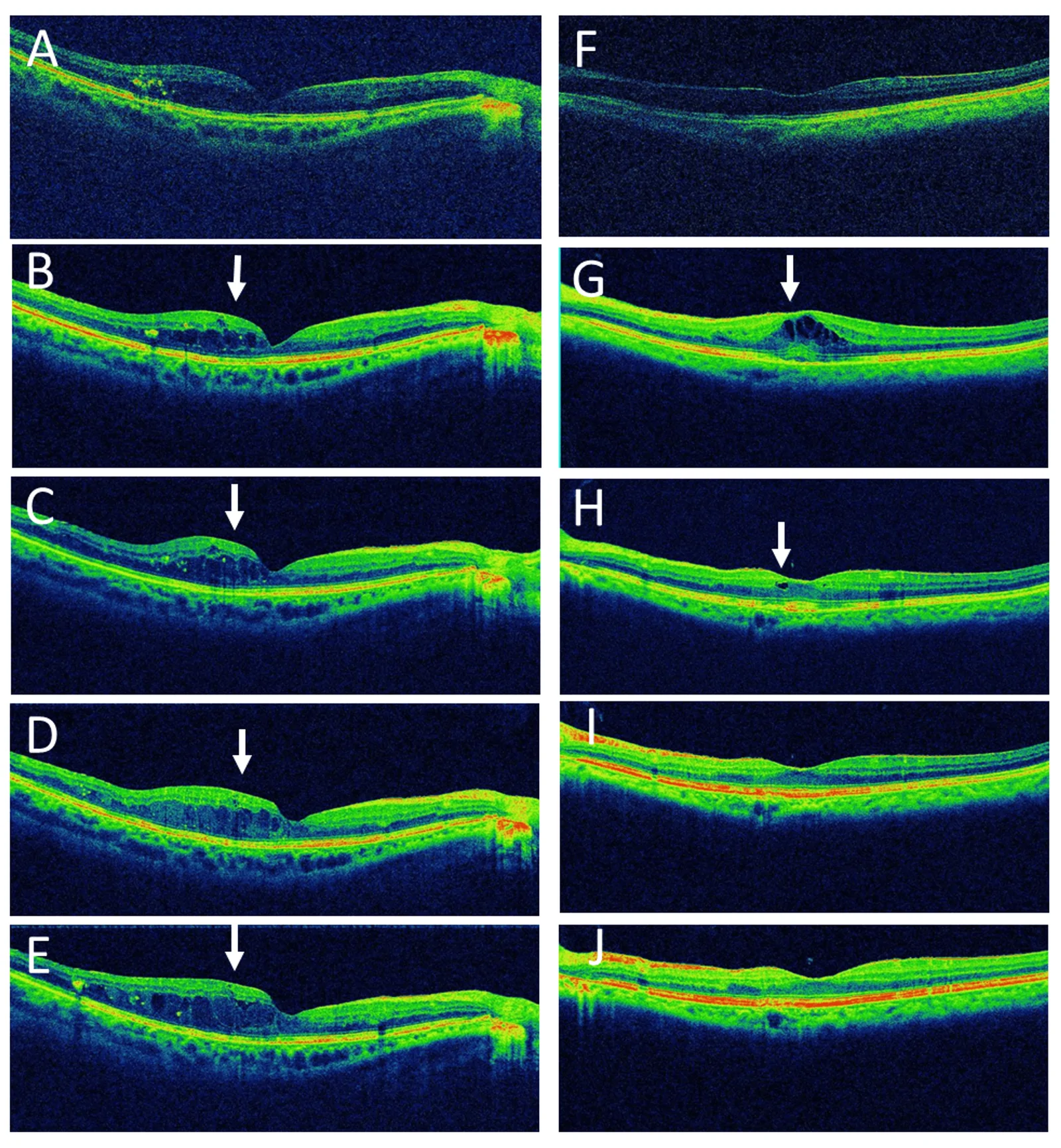
Optical coherence tomography in Cases 7 and 8. Case 7: Right eye of a 75-year-old man (A-E, left column). Horizontal cross-sectional optical coherence tomography images show no macular cyst before surgery (A) and temporal macular edema at 14 days (arrow, B), four months (arrow, C), seven months (arrow, D), and 10 months (arrow, E) after surgery. The postoperative regimen of topical 0.1% bromfenac and 0.1% fluorometholone was subsequently changed to topical 0.1% betamethasone. Case 8: Left eye of an 80-year-old woman (F-J, right column). Optical coherence tomography images show no macular cyst before surgery (F) and macular cysts 28 days after surgery while receiving topical 0.1% bromfenac alone (arrow, G). A small residual cyst was observed two weeks after the initiation of topical 0.1% betamethasone (arrow, H), with no cyst observed at one month (I) or eight months (J).

**Figure 6:**
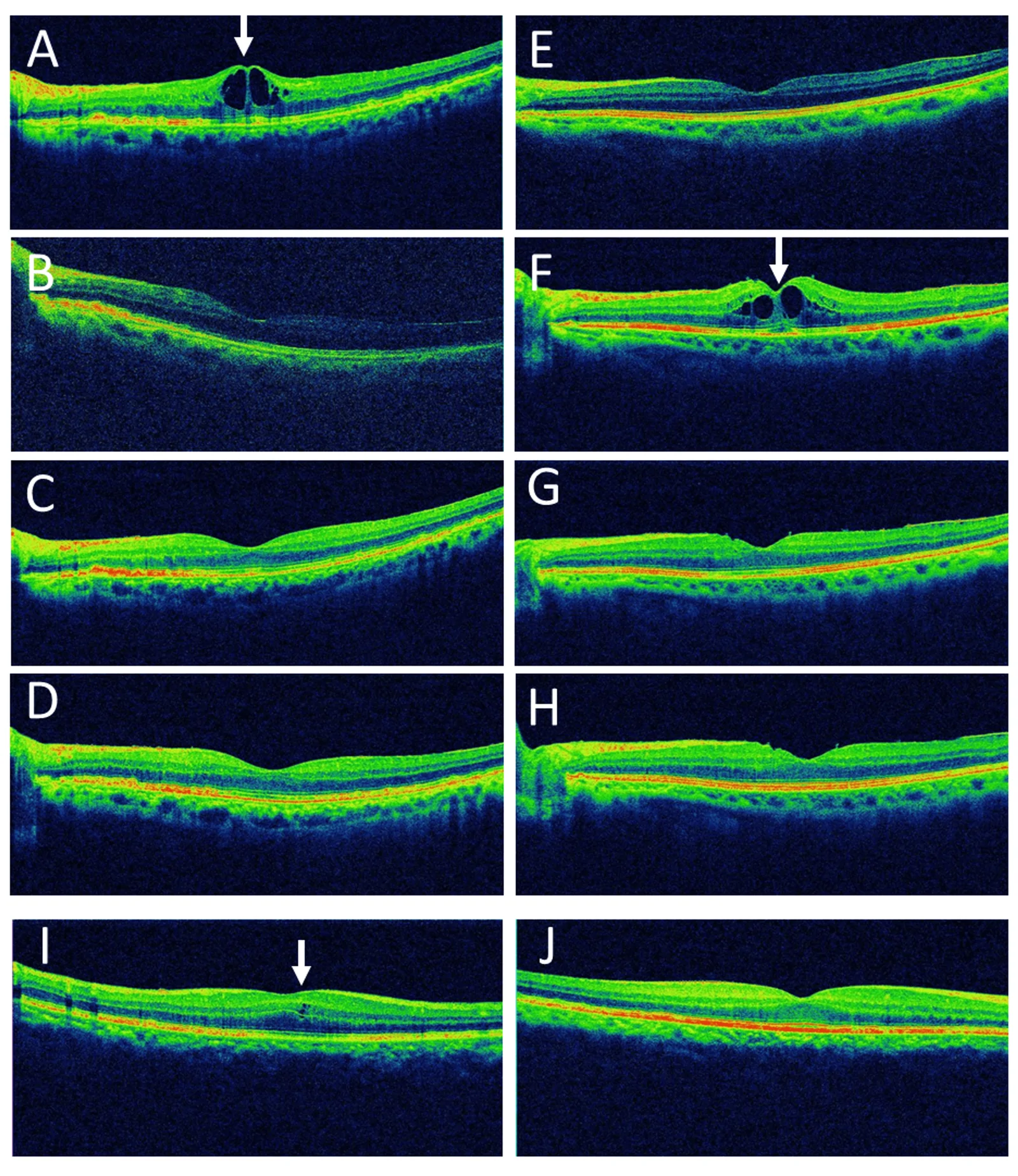
Optical coherence tomography in Cases 9, 10, and 11. Case 9: Left eye of a 74-year-old man (A-D, left column). Horizontal cross-sectional optical coherence tomography images show macular cysts five months after surgery following postoperative treatment with topical 0.1% bromfenac and 0.1% betamethasone (arrow, A). No cyst was observed at one month (B), two months (C), or four months (D) after the resumption of topical 0.1% betamethasone. Case 10: Left eye of a 78-year-old man (E-H, right column). Horizontal cross-sectional optical coherence tomography images show no macular cyst before surgery (E) and macular cysts two weeks after surgery while receiving topical 0.1% bromfenac alone (arrow, F). No cyst was observed at two weeks (G) or 1.5 months (H) after the initiation of topical 0.1% betamethasone. Case 11: Right eye of a 68-year-old man (I and J, bottom row). Vertical cross-sectional optical coherence tomography images show a small cyst one month after surgery while receiving topical 0.1% bromfenac alone (arrow, I) and no cyst one month after the initiation of topical 0.1% betamethasone (J).

**Figure 7:**
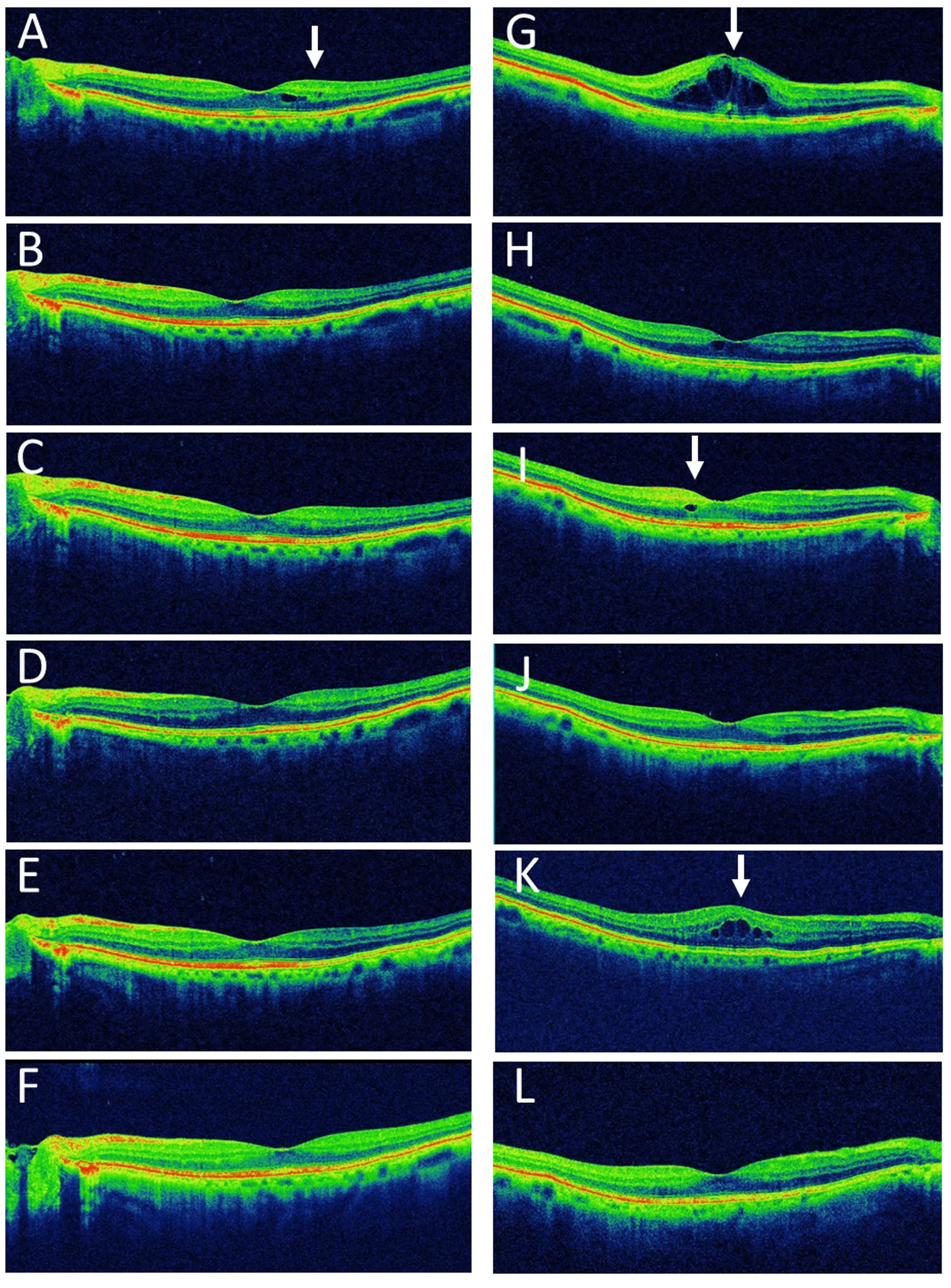
Optical coherence tomography in Case 12. Case 12: Left eye (A-F, left column) and right eye (G-L, right column) of an 80-year-old man. Cataract surgery was first performed on the left eye and, two weeks later, on the right eye. Each pair of images in the same row was obtained on the same date. Horizontal cross-sectional optical coherence tomography images show macular cysts in the left eye 28 days after surgery while receiving topical 0.1% bromfenac alone (arrow, A). No cyst was observed at three weeks (B), two months (C), three months (D), five months (E), or eight months (F) after the initiation of topical 0.1% betamethasone. In the right eye, macular edema was observed 14 days after surgery while receiving topical 0.1% bromfenac alone (arrow, G). Following treatment with topical 0.1% betamethasone, no cyst was observed at three weeks (H), a cyst was observed at two months (I), and no cyst was observed at three months (J). Macular cysts recurred at five months (K), but no cyst was observed at eight months (L).

**Figure 8:**
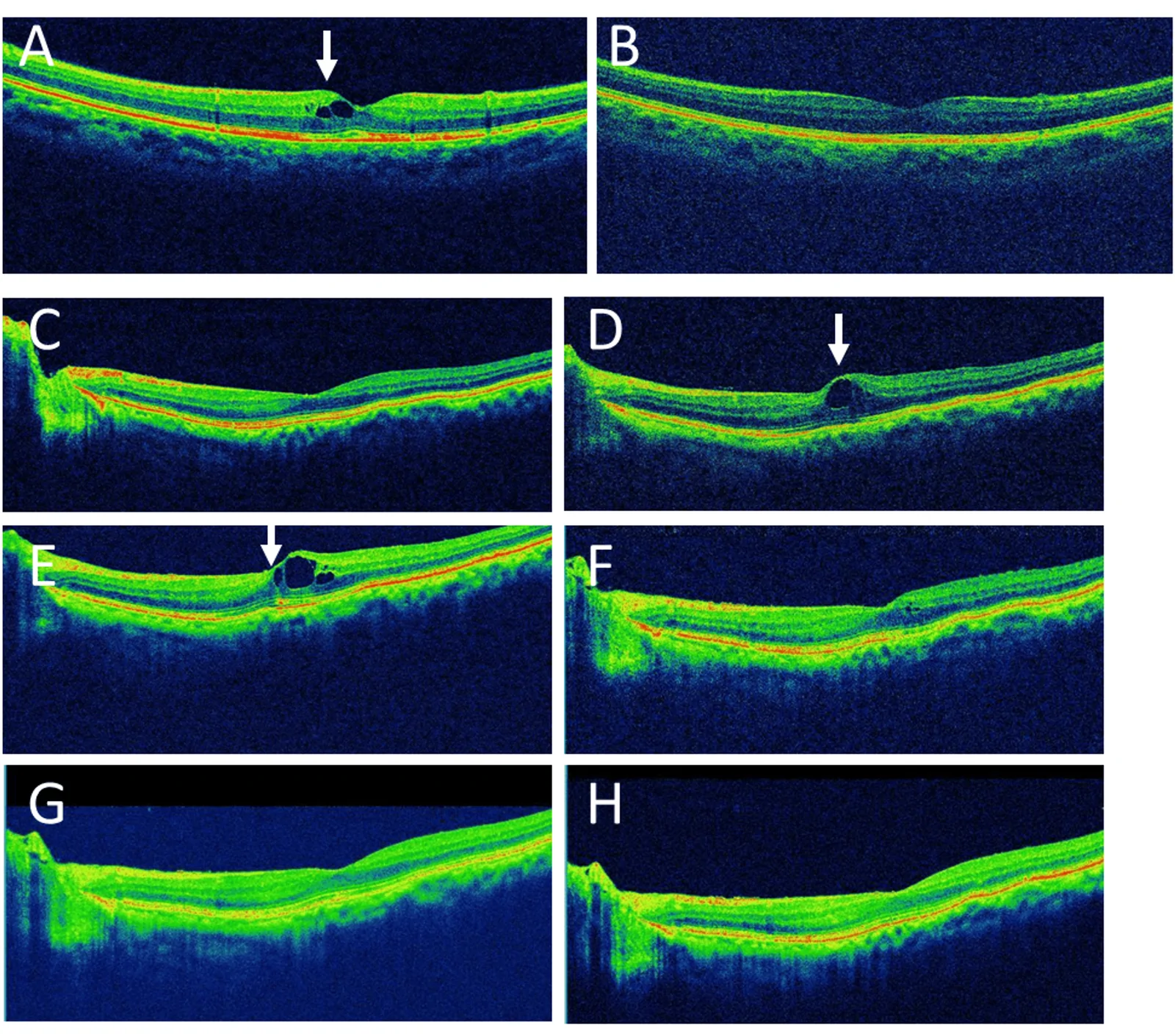
Optical coherence tomography in Cases 13 and 14. Case 13: Left eye of a 76-year-old man (A and B, top row). Vertical cross-sectional optical coherence tomography images show macular cysts two months after surgery following postoperative treatment with topical 0.1% bromfenac and 0.1% betamethasone (arrow, A) and no cyst one month after the resumption of topical 0.1% betamethasone (B). Case 14: Left eye of a 78-year-old woman (C-H). Horizontal cross-sectional optical coherence tomography images show no macular cyst seven days after surgery while receiving topical 0.1% bromfenac and 0.1% betamethasone (C) and macular cysts two months after surgery (arrow, D). Residual macular cysts remained three weeks after the resumption of topical 0.1% betamethasone (arrow, E). No cyst was observed at 1.5 months (F), 2.5 months (G), or four months (H) after resumption.

**Figure 9:**
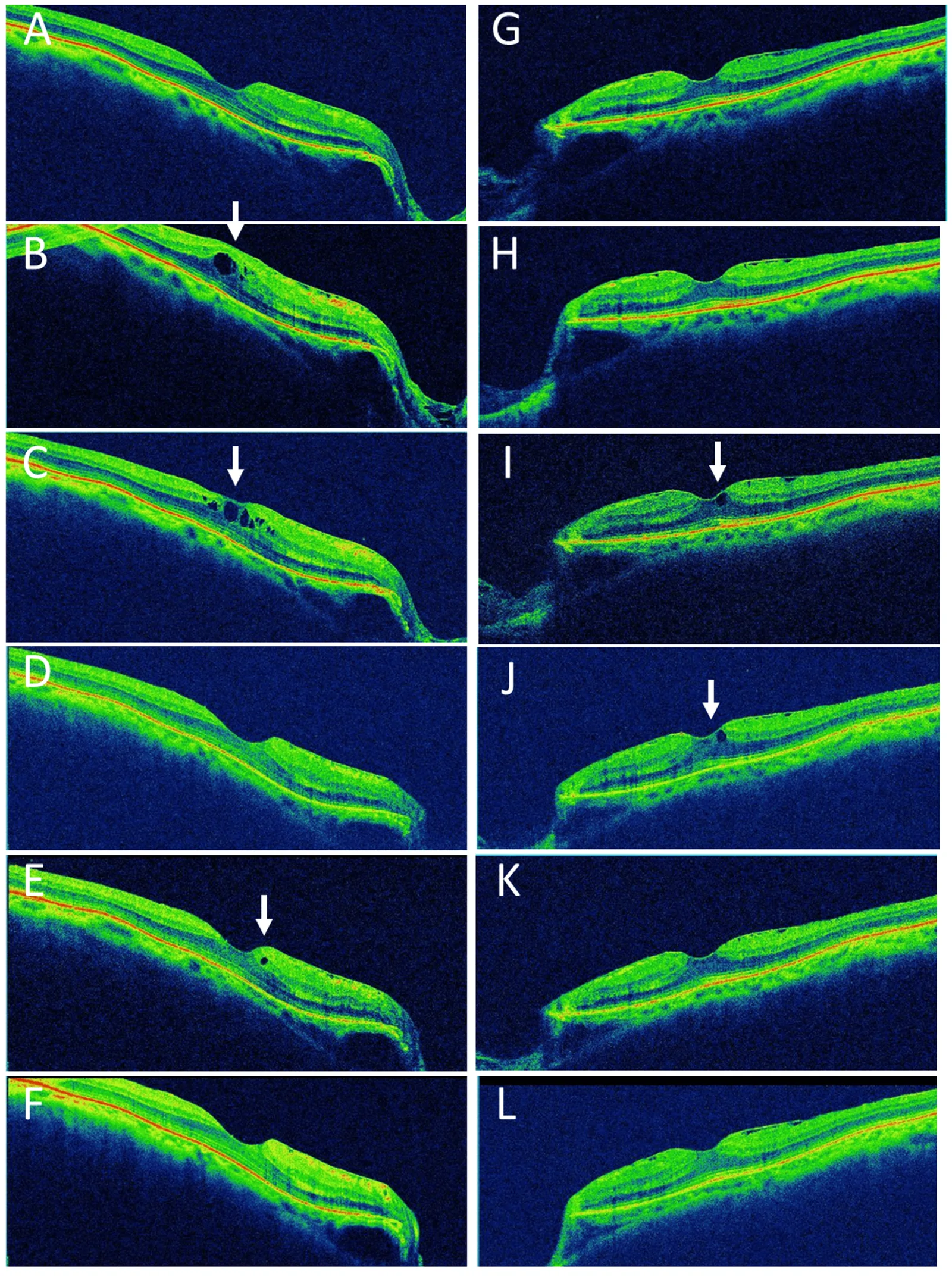
Optical coherence tomography in Case 15. Case 15: Right eye (A-F, left column) and left eye (G-L, right column) of a 71-year-old woman. Cataract surgery was first performed on the right eye and, two weeks later, on the left eye. Each pair of images in the same row was obtained on the same date. Horizontal cross-sectional optical coherence tomography images show no macular cyst in the right eye one month after surgery (A) and macular cysts two months after surgery following postoperative treatment with topical 0.1% bromfenac and 0.1% betamethasone (arrow, B). Residual macular cysts remained one month after the resumption of topical 0.1% betamethasone (arrow, C), whereas no cyst was observed at 1.5 months (D). A cyst recurred at four months (E), but no cyst was observed at five months after further treatment with topical 0.1% betamethasone (F). In the left eye, no macular cyst was observed at two weeks (G) or 1.5 months (H) after surgery following postoperative treatment with topical 0.1% bromfenac and 0.1% betamethasone. Note the minimal epiretinal membrane in the left eye. A cyst was observed eight weeks after surgery (arrow, I) and remained one month after the resumption of topical 0.1% betamethasone (arrow, J). No cyst was observed at three months (K) or four months (L) after treatment with topical 0.1% betamethasone.

**Figure 10:**
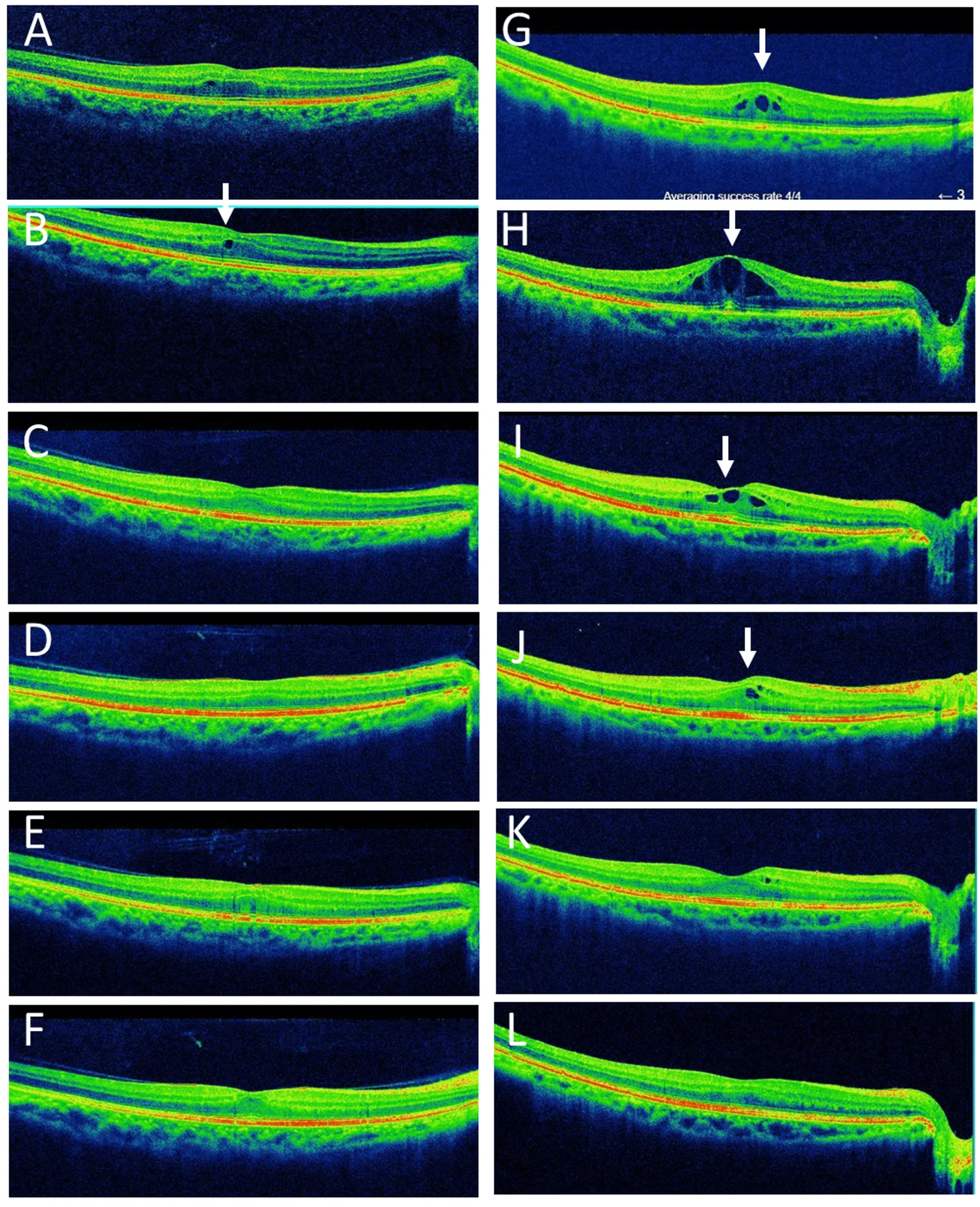
Optical coherence tomography in Cases 16 and 18. Case 16: Right eye of a 57-year-old man (A-F, left column). Horizontal cross-sectional optical coherence tomography images show no macular cyst 14 days after surgery (A) and a cyst five weeks after surgery following postoperative treatment with topical 0.1% bromfenac and 0.1% betamethasone (arrow, B). No cyst was observed at four weeks (C), two months (D), three months (E), or four months (F) after the resumption of topical 0.1% betamethasone. Case 18: Right eye of an 82-year-old woman (G-L, right column). Horizontal cross-sectional optical coherence tomography images show macular cysts one week after surgery while receiving topical 0.1% bromfenac alone (arrow, G). The macular edema increased one week after the initiation of topical 0.1% betamethasone (arrow, H). Macular cysts remained at three weeks (arrow, I), and a macular cyst remained at 1.5 months (arrow, J). No cyst was observed at five months (K) or nine months (L) after treatment with topical 0.1% betamethasone.

**Figure 11:**
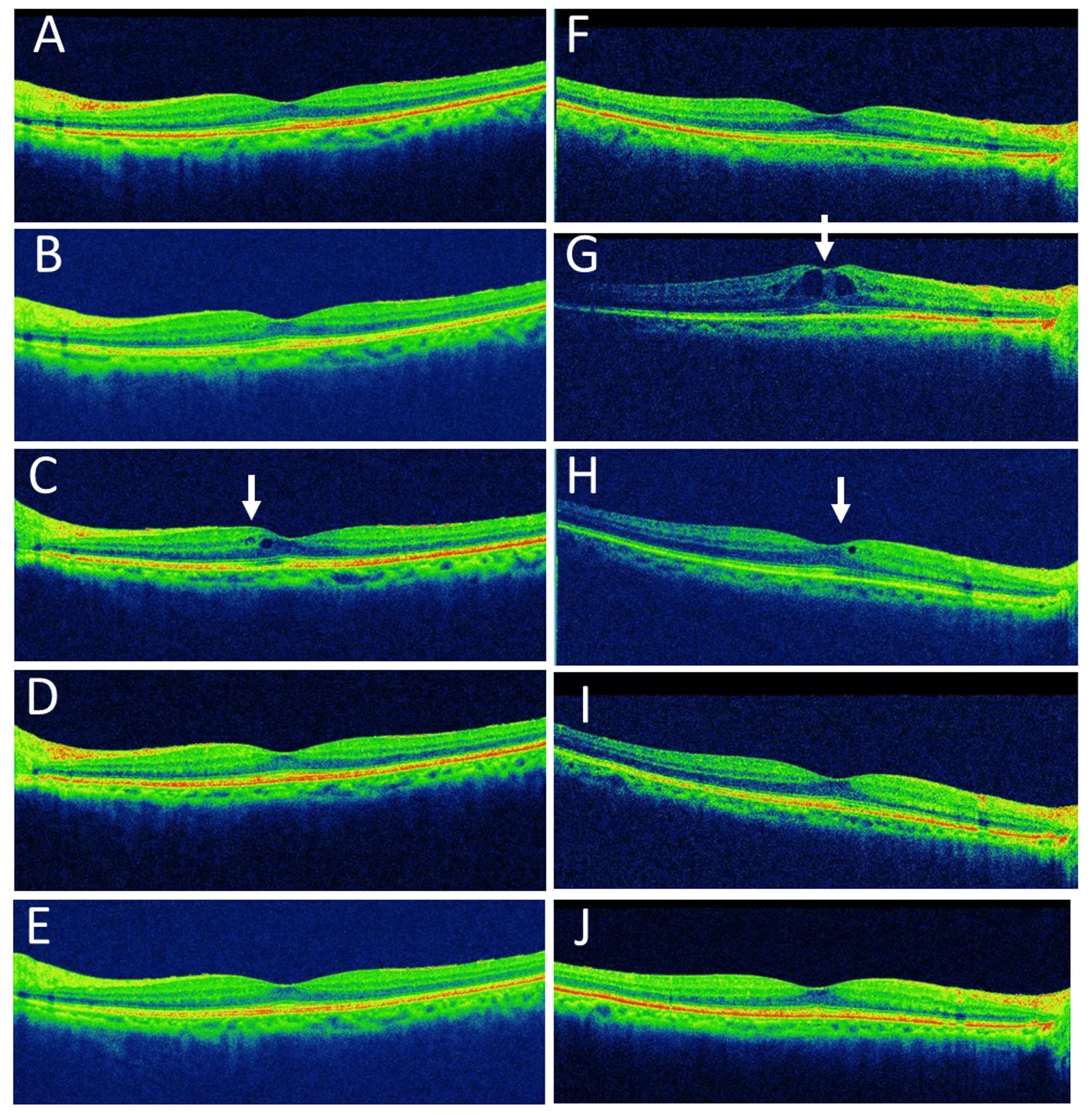
Optical coherence tomography in Case 17. Case 17: Left eye (A-E, left column) and right eye (F-J, right column) of an 82-year-old woman. Cataract surgery was first performed on the left eye and, two weeks later, on the right eye. Each pair of images in the same row was obtained on the same date. Horizontal cross-sectional optical coherence tomography images show no macular cyst in the left eye four weeks after surgery (A) or in the right eye two weeks after surgery (F) following postoperative treatment with topical 0.1% bromfenac and 0.1% betamethasone. No cyst was observed in the left eye two months after surgery (B), whereas macular cysts were observed in the right eye 1.5 months after surgery (arrow, G). A cyst was observed in the left eye three months after surgery (arrow, C), while a residual cyst remained in the right eye (arrow, H). Following the resumption of topical 0.1% betamethasone in both eyes, no cyst was observed in either eye at four months (left eye: D; right eye: I) or six months (left eye: E; right eye: J) after surgery.

**Figure 12:**
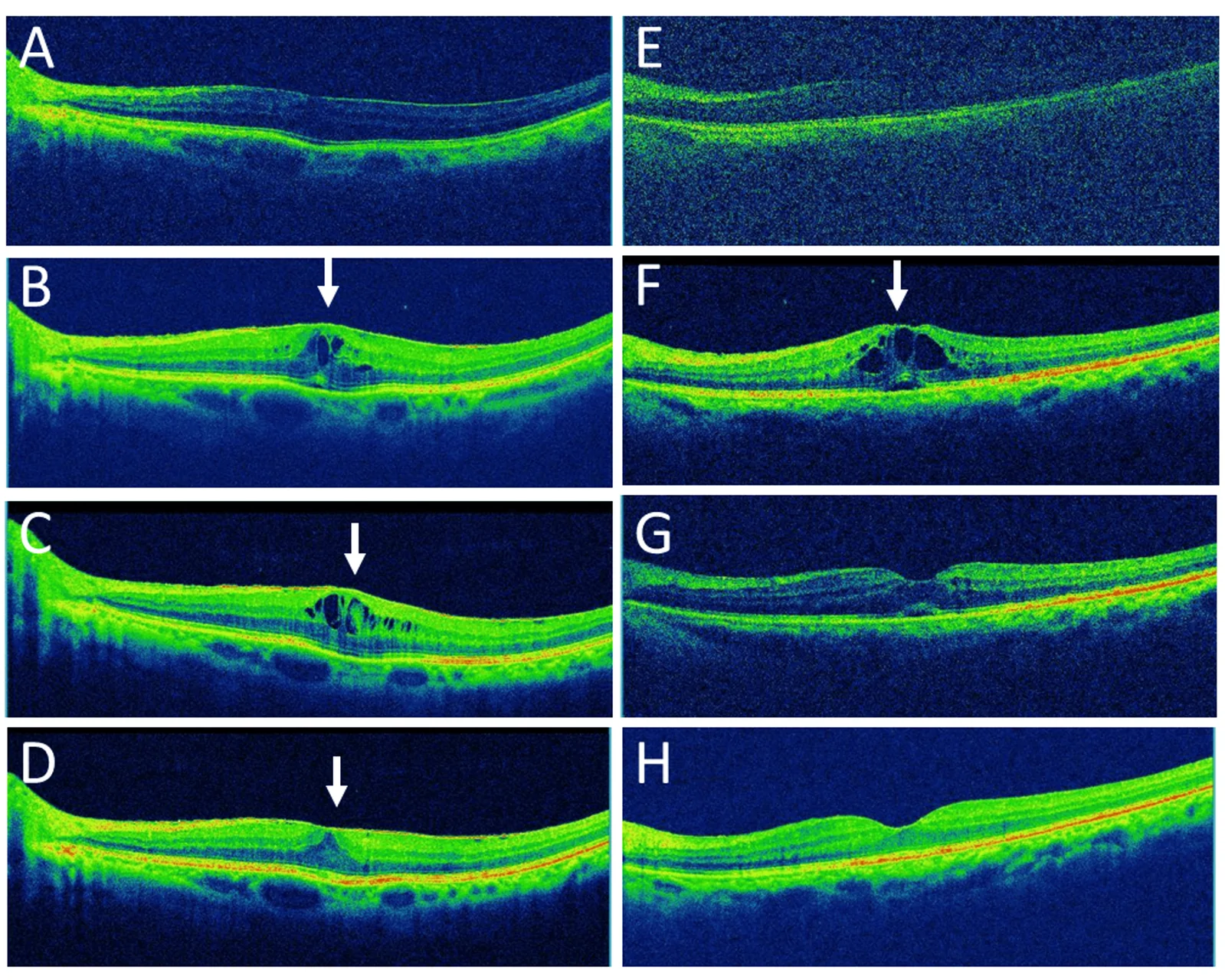
Optical coherence tomography in Cases 19 and 20. Case 19: Left eye of a 77-year-old man (A-D, left column). Horizontal cross-sectional optical coherence tomography images show no macular cyst before cataract surgery (A) and macular cysts two weeks after surgery while receiving topical 0.1% bromfenac alone (arrow, B). Macular cysts remained one month after the initiation of topical 0.1% betamethasone (arrow, C), but no cyst was observed at four months (D). Note the minimal epiretinal membrane in the left eye. Case 20: Left eye of a 77-year-old woman (E-H, right column). Horizontal cross-sectional optical coherence tomography images show no macular cyst before cataract surgery (E) and macular cysts one month after surgery following postoperative treatment with topical 0.1% bromfenac and 0.1% betamethasone (F). No macular cyst was observed at one month (G) or two months (H) after the resumption of topical 0.1% betamethasone. Images A and E are blurred because of the presence of cataracts before surgery.

**Figure 13:**
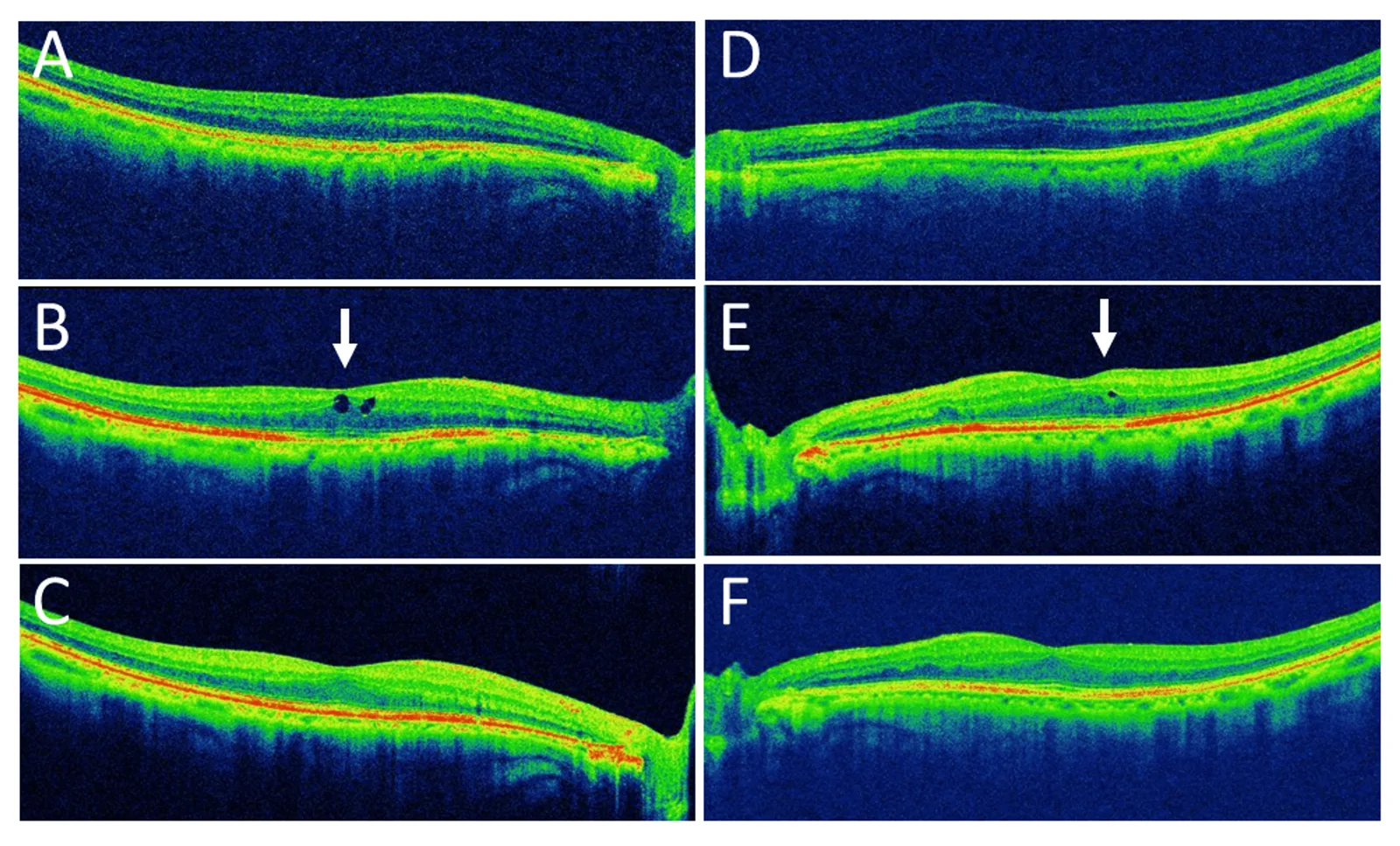
Optical coherence tomography in Case 22. Case 22: Right eye (A-C, left column) and left eye (D-F, right column) of a 77-year-old man. Cataract surgery was first performed on the right eye and, one week later, on the left eye. Each pair of images in the same row was obtained on the same date. Horizontal cross-sectional optical coherence tomography images show no macular cyst in the right eye (A) or left eye (D) before surgery. Macular cysts were observed in the right eye four weeks after surgery (arrow, B), and a small cyst was observed in the left eye three weeks after surgery (arrow, E), while the patient was receiving topical 0.1% bromfenac alone. No cyst was observed in either the right eye (C) or the left eye (F) one month after the initiation of topical 0.1% betamethasone.

Table 2 summarizes the clinical features of the 27 eyes of the 22 patients who developed macular edema after cataract surgery. Among the 1,325 consecutive operated eyes, one or more macular cysts were detected by optical coherence tomography in 27 eyes (2.0%) of 22 patients who complained of blurred vision or decreased visual acuity after having had no symptoms immediately after surgery (Table 2). The 22 patients (27 eyes: 12 right and 15 left) comprised 12 men and 10 women. Their ages at the time of surgery ranged from 57 to 82 years, with a median of 75.5 years. Of the 27 eyes that developed macular cysts, 13 eyes with no preoperative or intraoperative risk factors for inflammation received topical 0.1% bromfenac twice daily as the only anti-inflammatory eye drop, in combination with topical antibacterial 0.5% moxifloxacin four times daily. In contrast, the remaining 14 eyes, which had risk factors associated with inflammation, received topical bromfenac twice daily and 0.1% betamethasone (or 0.1% fluorometholone in two eyes) four times daily, as well as topical moxifloxacin.

**Table 2:** Clinical features of 27 eyes in 22 consecutive patients with pseudophakic cystoid macular edema (Irvine-Gass syndrome) identified among 1,325 consecutive cataract surgeries performed over four years (2022-2025). Bromfenac, 0.1% bromfenac sodium; betamethasone, 0.1% betamethasone sodium phosphate; fluorometholone, 0.1% fluorometholone; postop., postoperatively; ECCE: Extracapsular cataract extraction performed with intraocular lens implantation. *Cyst detection indicates the postoperative time point at which macular cysts were detected. BCVA: Best-corrected visual acuity; ECCE: Extracapsular cataract extraction; OCT: Optical coherence tomography.

Case no./sex	Age at surgery (years)	Laterality	Topical anti-inflammatory medication immediately after cataract surgery	Symptoms	Time from surgery to symptom onset or cyst detection	OCT findings	Ocular or surgical risk factors for macular edema	Systemic diseases and medications	Topical treatment and outcome	BCVA 5 days postop.	BCVA at cyst detection*	BCVA at final visit	Other features
1/Male (Figure 1)	74	Right	Bromfenac	Blurred vision	2 weeks	Macular cysts	None	Hypertension	Betamethasone; no macular cyst at 2 months	0.7	0.6	0.8	None
2/Male (Figure 1)	73	Right	Bromfenac, betamethasone	Blurred vision	2 months	Macular cysts	Exfoliation syndrome, latanoprost use	Hypertension, dyslipidemia, hyperuricemia	Resumption of betamethasone; no macular cyst at 1.5 months	1.2	1.2	1.2	Latanoprost was switched to timolol
3/Female (Figure 2)	72	Left	Bromfenac	Decreased visual acuity	1 month	Macular cysts	None	None	Betamethasone; no macular cyst at 3 weeks	0.7	0.6	1.2	Right-eye surgery 2 weeks later, with no signs of macular edema
4/Female (Figure 3)	74	Right	Bromfenac, betamethasone	Decreased visual acuity	2 months	Macular cysts	ECCE for mature cataract	Hypertension, dyslipidemia	Resumption of betamethasone; no macular cyst at 1 month	0.5	0.6	1.0	Macular cysts recurred 1 year later
4/Female (Figure 3)	74	Left	Bromfenac, betamethasone	Decreased visual acuity	2 weeks	Macular cysts	Minimal epiretinal membrane	Hypertension, dyslipidemia	Resumption of betamethasone; no macular cyst at 4 months	0.8	0.7	1.0	Right-eye surgery 1 month earlier
5/Female (Figure 4)	78	Right	Bromfenac	Decreased visual acuity	8 months	Macular cysts	None	Dyslipidemia	Betamethasone; no macular cyst at 7 months	1.2	0.5	0.8	Left-eye surgery 2 weeks later, with no signs of macular edema
6/Male (Figure 4)	74	Left	Bromfenac, fluorometholone	Blurred vision	13 months	Macular cysts	None	Pacemaker implantation; oral anticoagulant use	Betamethasone; no macular cyst at 1 month	1.0	0.4	0.8	None
7/Male (Figure 5)	75	Right	Bromfenac, fluorometholone	Metamorphopsia	2 weeks	Macular cysts	None; no diabetic retinopathy	Diabetes mellitus	Betamethasone; residual edema at 10 months	1.0	0.8	1.0	Left-eye surgery 2 weeks earlier, with no signs of macular edema
8/Female (Figure 5)	80	Left	Bromfenac	Blurred vision	1 month	Macular cysts	None	None	Betamethasone; no macular cyst at 1 month	0.8	0.7	1.0	Right-eye surgery 2 weeks later, with no signs of macular edema
9/Male (Figure 6)	74	Left	Bromfenac, betamethasone	Aniseikonia	5 months	Macular cysts	Concurrent pterygium resection	None	Resumption of betamethasone; no macular cyst at 1 month	0.8	0.3	0.8	Blunt trauma to the left eye at age 37
10/Male (Figure 6)	78	Left	Bromfenac	Blurred vision	2 weeks	Macular cysts	None	None	Betamethasone; no macular cyst at 2 weeks	1.0	0.7	0.8	Right-eye surgery 2 weeks later, with no signs of macular edema
11/Male (Figure 6)	68	Right	Bromfenac	Blurred vision	1 month	Macular cyst	High-grade hyperopia	Hypertension, benign prostatic hyperplasia	Betamethasone; no macular cyst at 1 month	1.2	0.5	0.8	Left-eye surgery 2 weeks earlier, with no signs of macular edema
12/Male (Figure 7)	80	Left	Bromfenac	Blurred vision	1 month	Macular cysts	None	Hypertension, dyslipidemia, hyperuricemia	Betamethasone; no macular cyst at 3 weeks	1.2	1.0	1.2	Right-eye surgery 2 weeks later
12/Male (Figure 7)	80	Right	Bromfenac	Blurred vision	2 weeks	Macular cysts (macular edema)	None	Hypertension, dyslipidemia, hyperuricemia	Betamethasone; no macular cyst at 3 weeks	0.5	0.4	1.0	Macular cysts recurred 1 and 4 months later
13/Male (Figure 8)	76	Left	Bromfenac, betamethasone	Blurred vision	2 months	Macular cysts	Poor mydriasis	Hypertension, chronic heart failure	Resumption of betamethasone; no macular cyst at 1 month	1.0	0.4	0.8	Right-eye surgery 1 week earlier, with no signs of macular edema
14/Female (Figure 8)	78	Left	Bromfenac, betamethasone	Blurred vision	2 months	Macular cysts	None	Hypertension	Resumption of betamethasone; no macular cyst at 1.5 months	0.8	0.4	1.0	Concurrent iritis at the onset of macular cysts; right-eye surgery 2 weeks later, with no signs of macular edema
15/Female (Figure 9)	71	Right	Bromfenac, betamethasone	Blurred vision	2 months	Macular cysts	Exfoliation syndrome, latanoprost use	None	Resumption of betamethasone; no macular cyst at 1.5 months	0.4	0.5	0.6	Latanoprost was switched to timolol; macular cysts recurred 4 months later
15/Female (Figure 9)	71	Left	Bromfenac, betamethasone	Blurred vision	2 months	Macular cysts	Exfoliation syndrome, latanoprost use, minimal epiretinal membrane	None	Resumption of betamethasone; no macular cyst at 3 months	0.5	0.4	0.6	Latanoprost was switched to timolol; right-eye surgery 2 weeks earlier
16/Male (Figure 10)	57	Right	Bromfenac, betamethasone	Decreased visual acuity	1 month	Macular cyst	None	Diabetes mellitus	Resumption of betamethasone; no macular cyst at 1 month	0.6	0.6	1.0	None
17/Female (Figure 11)	82	Left	Bromfenac, betamethasone	Blurred vision	3 months	Macular cyst	Poor mydriasis	None	Resumption of betamethasone; no macular cyst at 1 month	1.0	1.0	1.2	Right-eye surgery 2 weeks later
17/Female (Figure 11)	82	Right	Bromfenac, betamethasone	Blurred vision	1.5 months	Macular cysts	Poor mydriasis	None	Resumption of betamethasone; no macular cyst at 2 months	0.8	0.4	1.2	None
18/Female (Figure 10)	82	Right	Bromfenac	Decreased visual acuity	1 week	Macular cysts (macular edema)	None	Dyslipidemia	Betamethasone; no macular cyst at 3 months	0.5	0.5	1.0	None
19/Male (Figure 12)	77	Left	Bromfenac	Decreased visual acuity	2 weeks	Macular cysts	Minimal epiretinal membrane	Hypertension, dyslipidemia	Betamethasone; no macular cyst at 2 months	0.4	0.2	0.9	None
20/Female (Figure 12)	77	Left	Bromfenac, betamethasone	Decreased visual acuity	1 month	Macular cysts	Poor mydriasis	Hypertension, dyslipidemia	Resumption of betamethasone; no macular cyst at 1 month	1.2	1.0	1.0	Right-eye surgery 2 weeks later, with no signs of macular edema
21/Female (Figure 2)	72	Left	Bromfenac	Decreased visual acuity	2 weeks	Macular cyst	None	Dyslipidemia	Betamethasone; no macular cyst at 2 weeks	1.5	0.7	1.2	None
22/Male (Figure 13)	77	Right	Bromfenac	Blurred vision	4 weeks	Macular cysts	None	Hypertension	Betamethasone; no macular cyst at 2 weeks	1.2	1.2	1.2	Left-eye surgery 1 week later
22/Male (Figure 13)	77	Left	Bromfenac	No symptoms	3 weeks	Macular cyst	None	Hypertension	Betamethasone; no macular cyst at 2 weeks	1.2	1.2	1.2	None

The 13 eyes treated with topical bromfenac alone had no ocular risk factors, except for one eye with high-grade hyperopia. In contrast, among the 14 eyes treated with topical bromfenac and betamethasone (or fluorometholone in two eyes), the documented risk factors included exfoliation syndrome in three eyes, poor mydriasis in four eyes, planned extracapsular cataract extraction with intraocular lens implantation in one eye, concurrent pterygium resection in one eye, and diabetes mellitus without retinopathy in one eye. No risk factor was documented in four eyes. In this analysis, minimal epiretinal membranes, which were recognized only after the development of macular edema in three eyes, were not classified as risk factors.

The time from surgery to the diagnosis of macular cysts ranged from one week to eight months, with a median of two weeks, in the 13 eyes treated with topical bromfenac alone. In the 14 eyes initially treated with the combination of topical bromfenac and betamethasone (or fluorometholone), the time to diagnosis ranged from two weeks to 13 months, with a median of two months. In 26 of the 27 eyes, excluding one eye in Case 7, the macular cysts resolved and visual acuity returned to normal without residual symptoms within two weeks to seven months, with a median of one month, after topical betamethasone four times daily was initiated or resumed following its discontinuation. In the right eye of one patient (Case 7) with diabetes mellitus but no retinopathy, optical coherence tomography showed persistent low-level lamellar macular edema on the temporal side over a period of 10 months despite treatment with topical 0.1% betamethasone, although visual acuity remained good at 1.0.

## Discussion

The routine postoperative topical regimen after cataract surgery in the present series consisted of a NSAID and an antibacterial drug. In the presence of risk factors associated with inflammation, topical 0.1% betamethasone was added to the combination of topical NSAID and antibacterial eye drops. Under this clinical pathway for day surgery or overnight-stay surgery, pseudophakic cystoid macular edema, namely Irvine-Gass syndrome, had occasionally been encountered. Thus, the aim of this study was to determine the incidence and, particularly, the timing of the onset of pseudophakic cystoid macular edema in a consecutive series of cataract surgeries performed by a single surgeon at a single institution.

This study was designed beforehand to exclude cases of cystoid macular edema in patients with a history of branch retinal vein occlusion or the presence of diabetic retinopathy or a clinically significant macular epiretinal membrane because these retinal diseases could serve as apparent underlying causes of the onset or exacerbation of macular edema after cataract surgery [14]. However, the study did not exclude three eyes of three patients who developed pseudophakic cystoid macular edema in the presence of minimal epiretinal membranes that were recognized only retrospectively (the left eye of Case 4, the left eye of Case 15, and the left eye of Case 19). Cases of pseudophakic cystoid macular edema in patients with recent-onset uveitis were also excluded because uveitis is a known risk factor for pseudophakic cystoid macular edema, particularly when its onset is close to the time of cataract surgery or when it is not well controlled at the time of surgery [23-25].

In general, pseudophakic cystoid macular edema can be defined in either a narrow or broad sense, depending on whether ocular fundus diseases that are inherently prone to cause macular edema, irrespective of cataract surgery, are included [1, 2, 3, 4]. The present study adopted the narrow definition of pseudophakic cystoid macular edema.

Using this narrow definition, the present study identified two categories according to the time of onset of pseudophakic cystoid macular edema. The first category was the early onset of pseudophakic cystoid macular edema during the postoperative use of topical bromfenac alone in eyes with no preoperative, intraoperative, or postoperative risk factors. The second category was the late onset of pseudophakic cystoid macular edema after the initially prescribed topical betamethasone for risk factors was discontinued. The representative risk factors prompting the use of topical betamethasone in combination with topical bromfenac immediately after cataract surgery included exfoliation syndrome, poor mydriasis during surgery, and planned extracapsular cataract extraction with intraocular lens implantation. These clinical features are well-known risk factors for the development of pseudophakic cystoid macular edema [23-25]. It should be noted that only one of the 23 eyes with planned extracapsular cataract extraction and intraocular lens implantation developed pseudophakic cystoid macular edema after the initially prescribed postoperative topical betamethasone was discontinued (Tables 1-2). This finding suggests that extracapsular cataract extraction may not necessarily be a major risk factor for the development of pseudophakic cystoid macular edema.

Topical intraocular pressure-lowering prostaglandin analogues used to treat glaucoma, such as latanoprost, have been reported as risk factors for the development of cystoid macular edema, irrespective of cataract surgery [11, 12, 13]. In the present consecutive series of cataract surgeries performed over four years, prostaglandin analogues such as latanoprost were either temporarily suspended or continued depending on the intraocular pressure after cataract surgery. In the present study, three eyes of two patients receiving topical latanoprost for exfoliation glaucoma later developed pseudophakic cystoid macular edema after topical 0.1% betamethasone eye drops, prescribed immediately after cataract surgery, were discontinued, usually after two weeks. Following the onset of pseudophakic cystoid macular edema in these eyes, topical latanoprost was replaced with topical 0.5% timolol eye drops, and topical betamethasone eye drops were resumed.

Regarding the treatment of pseudophakic cystoid macular edema, topical 0.1% betamethasone eye drops administered four times daily were sufficient to achieve resolution of cystoid macular edema in all eyes except one (Case 7) [26, 27]. No patients required oral corticosteroids or sub-Tenon injections of long-acting corticosteroids such as triamcinolone. No patients returned with recurrent cystoid macular edema. As an exception, persistent lamellar macular edema was detected by optical coherence tomography for a prolonged period in one eye of one patient (Case 7), despite treatment with topical betamethasone, and may have been attributable to poorly controlled diabetes mellitus. No additional treatment was attempted because the patient maintained a decimal visual acuity of 1.0 in that eye.

During the first half of the four-year study period, topical 0.1% fluorometholone eye drops were used in combination with 0.5% moxifloxacin and 1% hyaluronate in patients with rheumatoid arthritis. During the second half of the study period, the postoperative topical regimen was changed to 0.1% betamethasone, 0.5% moxifloxacin, and 1% hyaluronate. The main reason for using these combinations was to avoid the postoperative use of topical bromfenac because topical or oral administration of NSAIDs may be associated with a risk of sterile corneal melting after cataract surgery [28, 29]. Topical 0.1% betamethasone was selected during the latter part of the study period because mild aqueous inflammation persisted in patients with rheumatoid arthritis who received topical 0.1% fluorometholone.

A major limitation of this retrospective study is that optical coherence tomography was not performed in all patients after cataract surgery. It was performed only when patients reported ocular symptoms or when their visual acuity was poorer than expected. The present study may therefore have missed patients with minimal macular cystic changes who remained asymptomatic. Another limitation is the clinical pathway itself, which was designed to prescribe topical 0.1% bromfenac as the baseline anti-inflammatory treatment and to add topical 0.1% betamethasone when conditions associated with an increased risk of inflammation or postoperative inflammation itself were present. The use of this clinical pathway was supported by the finding that approximately half of the operated eyes did not receive additional topical betamethasone after cataract surgery, as shown in the present study (Table 1). It should be noted that the hospital where this retrospective review was conducted is a hemodialysis center in the city. Consequently, the series included 32 (2.4%) cataract surgeries in patients undergoing hemodialysis (Table 1), which may represent a relatively high proportion. Hemodialysis did not appear to be a risk factor for the development of pseudophakic cystoid macular edema in the present study.

## Conclusions

Macular edema after cataract surgery is known as pseudophakic cystoid macular edema or Irvine-Gass syndrome. The aim of this study was to determine the incidence and timing of the onset of cystoid macular edema after cataract surgery. Among 1,325 consecutive eyes that underwent cataract surgery performed by a single surgeon at a single institution over four years (2022-2025), one or more macular cysts were detected by optical coherence tomography in 27 eyes (2.0%) of 22 patients. Of these eyes, 26 were associated with blurred vision or decreased visual acuity after having had no symptoms immediately after surgery, whereas one eye remained asymptomatic. The present study identified two categories according to the timing of the onset of pseudophakic cystoid macular edema. The first category was the early onset of pseudophakic cystoid macular edema during the postoperative use of topical bromfenac alone in eyes with no preoperative, intraoperative, or postoperative risk factors. The second category was the late onset of pseudophakic cystoid macular edema after the discontinuation of topical betamethasone that had initially been prescribed for risk factors in addition to routine topical bromfenac. Representative risk factors prompting the use of topical betamethasone in combination with topical bromfenac immediately after cataract surgery included exfoliation syndrome, poor mydriasis during surgery, and planned extracapsular cataract extraction with intraocular lens implantation.

Pseudophakic cystoid macular edema, namely Irvine-Gass syndrome, developed during the postoperative course in eyes with no preoperative or intraoperative risk factors that received topical bromfenac alone. The syndrome also developed later after the discontinuation of initially prescribed topical betamethasone in eyes with risk factors for inflammation. The macular cysts resolved following the initiation or resumption of topical betamethasone, accompanied by recovery of visual acuity and resolution of symptoms.
